# Effects of Ligands on Unfolding of the Amyloid β-Peptide Central Helix: Mechanistic Insights from Molecular Dynamics Simulations

**DOI:** 10.1371/journal.pone.0030510

**Published:** 2012-01-23

**Authors:** Mika Ito, Jan Johansson, Roger Strömberg, Lennart Nilsson

**Affiliations:** 1 Department of Biosciences and Nutrition, Karolinska Institutet, Huddinge, Sweden; 2 Department of Neurobiology, Care Sciences and Society (NVS) and Alzheimer Disease Research Center (KI-ADRC), Karolinska Institutet, Huddinge, Sweden; King's College London, United Kingdom

## Abstract

Polymerization of the amyloid β-peptide (Aβ), a process which requires that the helical structure of Aβ unfolds beforehand, is suspected to cause neurodegeneration in Alzheimer's disease. According to recent experimental studies, stabilization of the Aβ central helix counteracts Aβ polymerization into toxic assemblies. The effects of two ligands (Dec-DETA and Pep1b), which were designed to bind to and stabilize the Aβ central helix, on unfolding of the Aβ central helix were investigated by molecular dynamics simulations. It was quantitatively demonstrated that the stability of the Aβ central helix is increased by both ligands, and more effectively by Pep1b than by Dec-DETA. In addition, it was shown that Dec-DETA forms parallel conformations with β-strand-like Aβ, whereas Pep1b does not and instead tends to bend unwound Aβ. The molecular dynamics results correlate well with previous experiments for these ligands, which suggest that the simulation method should be useful in predicting the effectiveness of novel ligands in stabilizing the Aβ central helix. Detailed Aβ structural changes upon loss of helicity in the presence of the ligands are also revealed, which gives further insight into which ligand may lead to which path subsequent to unwinding of the Aβ central helix.

## Introduction

Alzheimer's disease (AD) is one of the most common neurodegenerative disorders in aging people. According to the amyloid cascade hypothesis [1], [2], [3], accumulation of the amyloid β-peptide (Aβ) in the brain is the primary influence driving AD pathogenesis. Originally insoluble fibrils and plaques composed of Aβ were suspected to cause AD [1], [2], but currently prefibrillar aggregates including soluble oligomers composed of Aβ are also considered to be the cause of AD [3]. Aβ is produced mainly as a 40- or 42-residue peptide by proteolysis of an integral membrane protein, the amyloid precursor protein (APP). Nuclear magnetic resonance (NMR) data showed that Aβ(1–40) adopts a folded structure including two α-helical regions (residues 15–24 and 29–35) in water/sodium dodecyl sulfate (SDS) micelles which provide a water-membrane interface mimicking environment [4], [5], and that Aβ(1–42) adopts an unfolded structure including two β-strands (residues 17–21 and 31–36) in aqueous solution [6]. Using NMR it has also been shown that an Aβ(1–42) fibril is a β-sheet composed of two β-strands (residues 18–26 and 31–42) [7]. These structural data indicate that, once Aβ departs from the membrane to the extracellular fluid, its α-helical regions unfold to elongated or β-strand-like forms, and that the β-strands of Aβ enable formation of β-sheets of fibrils and prefibrillar aggregates.

A wide range of molecules including small compounds and synthetic peptide derivatives have been identified as anti-amyloid agents [8]. Most of these molecules are predicted to bind to elongated or β-strand-like Aβ and to inhibit β-sheet extension, and thus they are expected to prevent Aβ polymerization. However, this strategy may be problematic in that it will favor formation of prefibrillar aggregates such as Aβ oligomers which are cytotoxic [9], and that some of the ligands may act as aggregators [10]. Alternative strategies to develop anti-amyloid agents are needed to overcome these problems. Earlier steps in amyloidogenesis before emergence of β-strand-like Aβ should be targeted to pursue alternative strategies. The emergence of β-strand-like Aβ can be inhibited by trapping Aβ in a state similar to its native structure in membrane embedded APP.

Recent experimental studies [11], [12] demonstrated that trapping Aβ in a state similar to its native structure by stabilizing the Aβ central helix (residues 15–24) is an effective strategy to reduce Aβ polymerization and Aβ toxicity. Two different classes of ligands were designed to bind and stabilize the Aβ central helix, and it was shown that in the presence of either ligand, Aβ helical content was increased, the amount of Aβ fibrils was reduced, Aβ toxicity to PC12 cells in culture and to hippocampal slice preparations was reduced, and the lifespan of Drosophila model was prolonged [12]. Although many effects of the two ligands (Dec-DETA and Pep1b) are similar, there are also different effects on polymerization. That is, thicker-than-normal Aβ fibrils were detected in the presence of Dec-DETA, and shorter-than-normal Aβ fibrils were detected in the presence of Pep1b, though both ligands substantially reduced the amount of Aβ fibrils. The reason for this was not clarified in the experimental study. We suspect that there are differences in behavior toward Aβ between the two ligands.

In order to rationally design new compounds that more effectively stabilize the Aβ central helix and reduce Aβ polymerization into toxic assemblies, detailed molecular mechanisms that underlie unfolding and stabilization of the Aβ central helix should be elucidated. Elucidation of such detailed molecular mechanisms, which are difficult to analyze by using only experimental methods, is possible by taking advantage of computational methods like molecular dynamics (MD). The unfolding process of the Aβ helix has attracted much attention and has been studied by MD simulations [13], [14], [15], [16], [17]. However, effects of ligands on the unfolding process of the Aβ helix have not been fully investigated and detailed molecular mechanisms for the Aβ helix stabilization by ligands have not been uncovered yet, though short MD simulations indicated that the designed ligands stabilize the α-helical conformation of Aβ(13–26) [12].

In the present study, effects of the two ligands (Dec-DETA and Pep1b) which were designed in the previous experimental study [12] on the unfolding process of the Aβ central helix (residues 15–24) were investigated by MD simulations. The middle region (residues 15–24) of Aβ is of interest, because a short Aβ(16–20) fragment included in this region is capable of binding to full-length Aβ [18], [19] and to the fragment itself [20], [21], in addition, stabilization of this region in an α-helical conformation by mutations or by ligands counteracts Aβ polymerization into toxic assemblies [11], [12]. For the present study, we performed MD simulations for α-helical Aβ(13–26) in the absence or presence of either ligand, since our previous study [17] showed that MD simulations for the short peptide Aβ(13–26) represent the difference between wild-type Aβ and its mutants in good agreement with experimental data. Here we demonstrate that the two ligands are effective in stabilizing the Aβ central helix in agreement with experiments, and thereupon, we compare effects of the two ligands on unwinding of the Aβ central helix. Furthermore, we suggest a possible explanation to why the lower amount of fibrils formed from unwound Aβ monomers incubated with Dec-DETA and with Pep1b are thicker-than-normal and shorter-than-normal, respectively.

## Methods

### Preparation of Systems

An initial model structure of Aβ(13–26), whose sequence is HHQKLVFFAEDVGS, was built as an α-helix using the Insight II program (version 2000) [22], because the middle region (residues 15–24) of the full-length Aβ adopts an α-helical conformation surrounded by flexible unstructured regions in a water-membrane interface mimicking environment as shown by experimental studies [4], [5]. Available NMR structures (entry 1BA4 [4] in the Protein Data Bank [23]) of the full-length Aβ have eight or nine backbone O(*i*)-HN(*i*+4) hydrogen bonds in the fourteen-residue region (residues 13–26), including six backbone O(*i*)-HN(*i*+4) hydrogen bonds in the middle region (residues 15–24). We therefore built the whole peptide (residues 13–26) as an α-helix which has ten backbone O(*i*)-HN(*i*+4) hydrogen bonds. Since Aβ(13–26) is a fragment of the full-length Aβ, the N- and C-termini of our model were capped with N-terminal acetyl and C-terminal amide groups, respectively, mimicking the uncharged amide linkage that is adjacent to Aβ(13–26) on both ends in the full-length Aβ.

**Figure 1:**
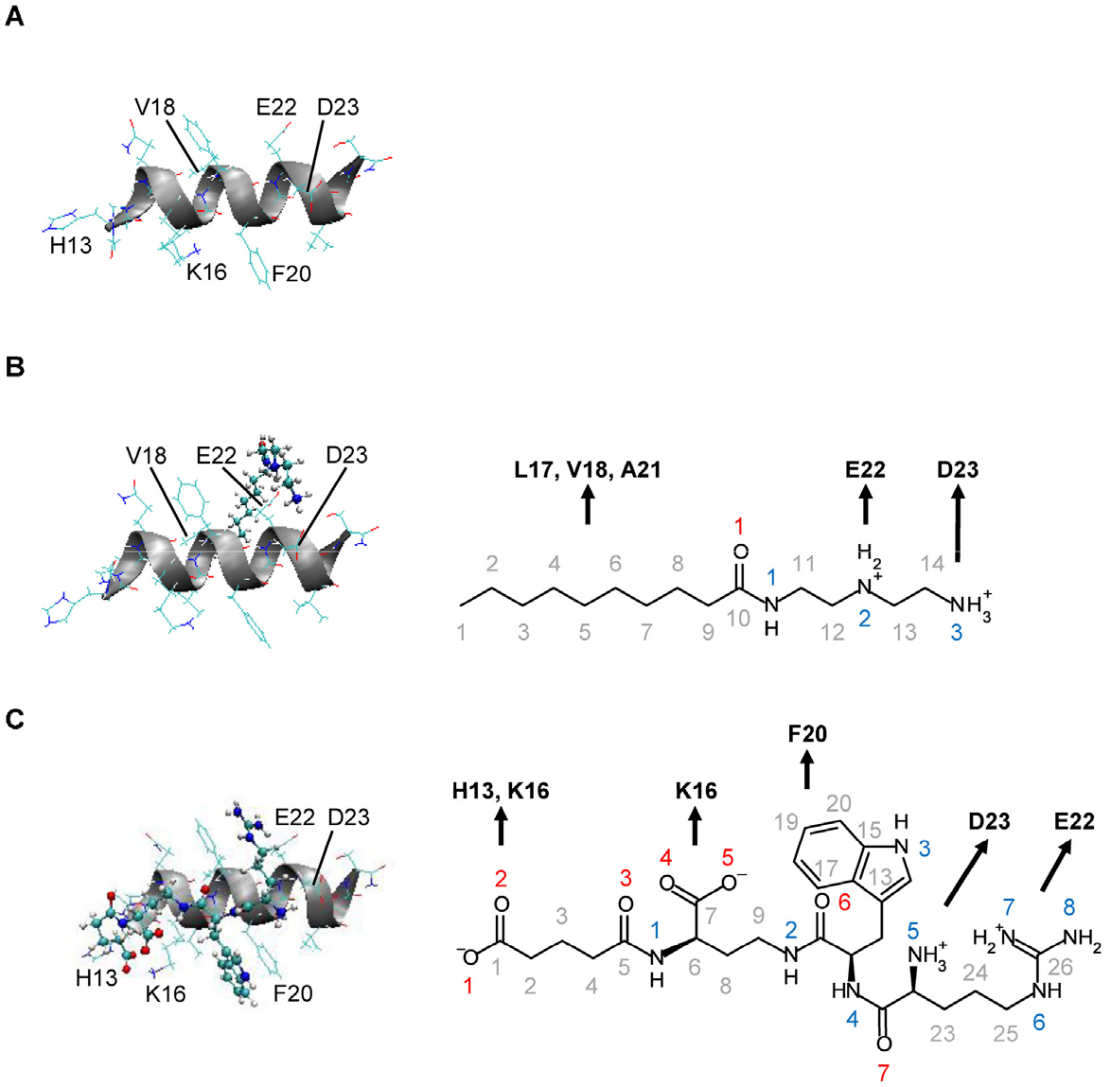
Initial structures of Aβ and the Aβ-ligand complexes. The initial energy-minimized structures of Aβ (A), the Aβ-Dec-DETA complex (B), and the Aβ-Pep1b complex (C) are shown. The positions of Aβ backbones (ribbons), Aβ sidechains (lines), and the ligands (lines and balls) are displayed. The structural formulae of Dec-DETA (B) and Pep1b (C) are also shown. The numbering of carbon (gray), nitrogen (blue), and oxygen (red) atoms is indicated. The residues of Aβ with which the different groups of the ligands are designed to interact (arrows) are also indicated.

Structures of the two ligand-peptide complexes were manually built using the Insight II program to satisfy the ligand-peptide contacts that were intended in their design: The Dec-DETA complex was designed for electrostatic interaction with E22 and D23 via the two basic functional groups and for van der Waals interaction with L17, V18, and A21 via the hydrocarbon tail (Fig. 1); similarly the Pep1b complex was built for electrostatic interaction with E22 and D23 via the two basic functional groups and with H13 and K16 via the two acidic functional groups, and for van der Waals interaction with F20 via the indole group (Fig. 1).

According to an NMR structure (entry 1HZ3 [24] in the Protein Data Bank [23]) of the unfolded Aβ in water at pH 5.7, all the ionizable residues are in their charged states. Besides, in our previous study [17], we showed that similar results were obtained for the Aβ models regardless of the histidine protonation states. Therefore, all the ionizable residues of Aβ(13–26) were prepared in their charged states, where the basic residues (H13, H14, and K16) were protonated at the sidechain N atoms and the acidic residues (E22 and D23) were deprotonated at the sidechain O atoms. All the ionizable functional groups of Dec-DETA and Pep1b were also prepared in their charged states. The total charges of Aβ(13–26), the Aβ(13–26)-Dec-DETA complex, and the Aβ(13–26)-Pep1b complex are +1*e*, +3*e*, and +1*e*, respectively; the systems were neutralized by adding 1, 3, or 1 chloride counterions. Each model was solvated in a rhombic dodecahedron water box filled with TIP3P [25] water molecules with a minimum solute-wall distance of 10 Å. Water molecules with the oxygen atom less than 2.2 Å from any heavy peptide atom were deleted, and 3028, 3021, and 3007 water molecules remained in the Aβ(13–26), Aβ(13–26)-Dec-DETA, and Aβ(13–26)-Pep1b systems, respectively.

### MD Simulations

All calculations were carried out using the CHARMM22/CMAP force field [26], [27], [28] with the CHARMM program [29], [30]. The force field parameters for the ligands (Table S1) were picked from the CHARMM22 force field parameters for proteins, since the ligands were designed basically using amino acid moieties. The SHAKE [31] algorithm was applied to fix all covalent bonds containing a hydrogen atom allowing a 2 fs timestep to be used in the integration of Newton's equations. The nonbonded (van der Waals and Coulomb) interaction energies and forces were smoothly shifted to zero at 12 Å using the atom-based force-shift method [32], [33], and the nonbonded list was constructed with a cutoff of 16 Å and was updated every time any atom moved by more than 2 Å since the last update. Before MD simulations were carried out, structures of the solvated systems were optimized by 500 steps of steepest descent energy minimization with a harmonic restraint of 20 kcal/mol/Å^2^ on Aβ followed by 1500 steps of adopted basis Newton-Raphson energy minimization without a harmonic restraint on Aβ (Fig. 1). After the systems were heated up to 360 K gradually for 50 ps, ten independent 20 ns MD simulations at 360 K with different initial velocity assignments were carried out for each system to increase sampling [34]. The MD simulations were performed for the optimized systems under periodic boundary conditions at a constant pressure (1 atm) using the Langevin piston method [35] with piston mass 400 amu, collision frequency 20 ps^−1^ and bath temperature (360 K). The average temperature was checked every 4 ps, and was found to remain within 5 K of the target temperature after the heating MD run. Fast table lookup routines for non-bonded interactions [36] were used to increase speed of the MD simulations. During the MD simulations, no harmonic restraints were imposed on any molecule in the systems, and coordinates were saved every 1 ps.

In our previous study [17], we showed that the Aβ central helix completely unfolded at 360 K in 20 ns MD simulations, though it did not unfold at the lower temperatures (300 and 330 K). Therefore, the simulations for each system were performed at 360 K to accelerate dynamics of Aβ. Additionally, one control 20 ns MD simulation for each system was performed at 310 K with the methods used for the MD simulations at 360 K.

### Analyses

All analyses were carried out for the trajectories obtained by the MD simulations at 360 K, except as otherwise stated. The data of every 10 ps of the trajectories after the heating time of the MD simulations were used for the analyses. Visualization of the structural change of the Aβ and Aβ-ligand complex models during MD simulations was carried out by using the visual molecular dynamics (VMD) software (version 1.8.6) [37].

To examine the structural change of Aβ quantitatively, the root-mean-square deviation (RMSD) and radius of gyration (R_g_) were calculated for the middle region (15–24) of Aβ(13–26), thus large fluctuations of the RMSD and R_g_ due to the mobile N- and C-termini were eliminated. Before the RMSD measurements, overall rotation and translation were removed by least-squares superposition using coordinates of all heavy atoms of the initial energy-minimized Aβ structure obtained prior to the MD simulations. The RMSD was calculated for backbone heavy atoms against the initial energy-minimized coordinates and the R_g_ was calculated for all atoms along the MD simulation time.

To discriminate the type or the pattern of the Aβ structure, the number of α-helical O(*i*)-HN(*i*+4) backbone hydrogen bonds (αHBs) in the middle region (15–24) was calculated, using the criterion acceptor-hydrogen distance ≤2.4 Å to define the existence of a hydrogen bond [38].

To examine how each ligand interacted with Aβ during the simulations, the probability of the contact between the center of geometry of sidechain heavy atoms of each Aβ residue and each ligand heavy atom was calculated, using the criterion distance ≤6.0 Å. The distance criterion (6.0 Å) was chosen considering the contact distances measured for the initial energy-minimized structures of the Aβ-ligand complexes. A map of the contacts between Aβ and Dec-DETA or Pep1b was created using the calculated probabilities.

To determine details of polar interactions between Aβ and each ligand, the number of hydrogen bonds (HBs) between Aβ and Dec-DETA or Pep1b was calculated, using the criterion acceptor-hydrogen distance ≤2.4 Å. For this calculation, both of HBs between Aβ sidechain atoms and ligand atoms and HBs between Aβ backbone atoms and ligand atoms were counted (for each Aβ-ligand complex, the number of the latter HBs was less than 10% of that of all the HBs). When at least one HB between Aβ and the ligand was observed, the ligand was considered to be bound to Aβ.

Additionally, to determine details of nonpolar interactions between Aβ and each ligand, the number of C-C and C-N contacts between carbon atoms of the Aβ middle nonpolar part (residues 17–21) and nine heavy atoms of the Dec-DETA hydrocarbon tail (C1–C9) or of the Pep1b indole group (C13–C20 and N3) was calculated, using the criterion C-C or C-N distance ≤5.0 Å. The backbone carbonyl carbon atoms of Aβ were not included in this calculation. The distance criterion (5.0 Å) was chosen considering the radii of carbon (1.8–2.3 Å), nitrogen (1.9 Å), and hydrogen (1.3–1.4 Å) atoms and the C-H and N-H covalent bond lengths (1.0–1.1 Å) used in the CHARMM22 force field [26]. When at least one contact between the Aβ middle nonpolar part and the ligand nonpolar part was observed, the ligand nonpolar part was considered to be in contact with the Aβ middle nonpolar part. In this analysis, contacts between the Aβ middle nonpolar part and the ligand nonpolar parts (the hydrocarbon tail of Dec-DETA and the indole group of Pep1b) were focused on, because it was shown that, during the simulations, the ligand nonpolar parts were mainly in contact with the Aβ middle nonpolar part as they were designed (Fig. 1).

## Results

### Effects of the Ligands on Stability of the Aβ Central Helix

To examine whether the Aβ central helix eventually unfolded by the end of the simulation, the average backbone RMSD of the Aβ middle region (15–24) and the average number of αHBs of the Aβ middle region calculated for the last 2 ns of the each 20 ns simulation, where fluctuation of the Aβ backbone RMSD is relatively small in every trajectory, were analyzed (Table 1). The trajectories were classified into three groups: group A (RMSD<2.0 Å, 2≤αHB≤6), group B (2.0 Å≤RMSD<4.0 Å, 1≤αHB≤4), and group C (RMSD≥4.0 Å, αHB≈0). By visual inspection, it was ascertained that the Aβ central helix maintained its helical conformation during the whole simulations or refolded after partial unfolding by the end of the simulations in the group A trajectories, that it partially unfolded by the end of the simulations in the group B trajectories, and that it completely unfolded by the end of the simulations in the group C trajectories. The helical Aβ (group A) is observed in only one trajectory in the absence of a ligand, whereas it is observed in five trajectories in the presence of Dec-DETA and is observed in four trajectories in the presence of Pep1b (Table 1). In contrast, the completely unfolded Aβ (group C) is observed in three trajectories in the absence of a ligand, whereas it is observed in only one trajectory in the presence of Dec-DETA and is not observed in any trajectory in the presence of Pep1b (Table 1).

**Table 1:** Average RMSD (Å) and average number of αHBs during the last 2 ns of the 20 ns MD simulations calculated for the Aβ middle region in the absence or presence of Dec-DETA or Pep1b.

	average RMSD			average number of αHBs			group^a^		
trajectory	no ligand	Dec-DETA	Pep1b	no ligand	Dec-DETA	Pep1b	no ligand	Dec-DETA	Pep1b
1	2.59	1.09	0.91	2.4	3.5	3.6	B	A	A
2	2.79	2.23	1.79	2.8	3.1	2.5	B	B	A
3	2.20	3.78	3.73	2.5	0.8	0.8	B	B	B
4	5.25	5.50	0.91	0.0	0.1	4.4	C	C	A
5	3.84	2.55	3.65	0.9	1.9	1.6	B	B	B
6	2.29	1.51	2.21	1.8	3.6	3.7	B	A	B
7	4.85	1.46	1.38	0.4	4.5	2.3	C	A	A
8	3.06	1.36	2.12	1.4	4.2	2.3	B	A	B
9	4.89	0.85	3.07	0.1	4.3	2.3	C	A	B
10	1.24	3.18	2.21	4.2	3.7	3.4	A	B	B
mean value	3.30	2.35	2.20	1.6	3.0	2.7			
SD	1.35	1.45	1.02	1.3	1.5	1.1			

aThe trajectories are classified into three groups: A) RMSD<2.0 Å and 2≤αHB≤6, B) 2.0 Å≤RMSD<4.0 Å and 1≤αHB≤4, and C) RMSD≥4.0 Å and αHB≈0.

**Figure 2:**
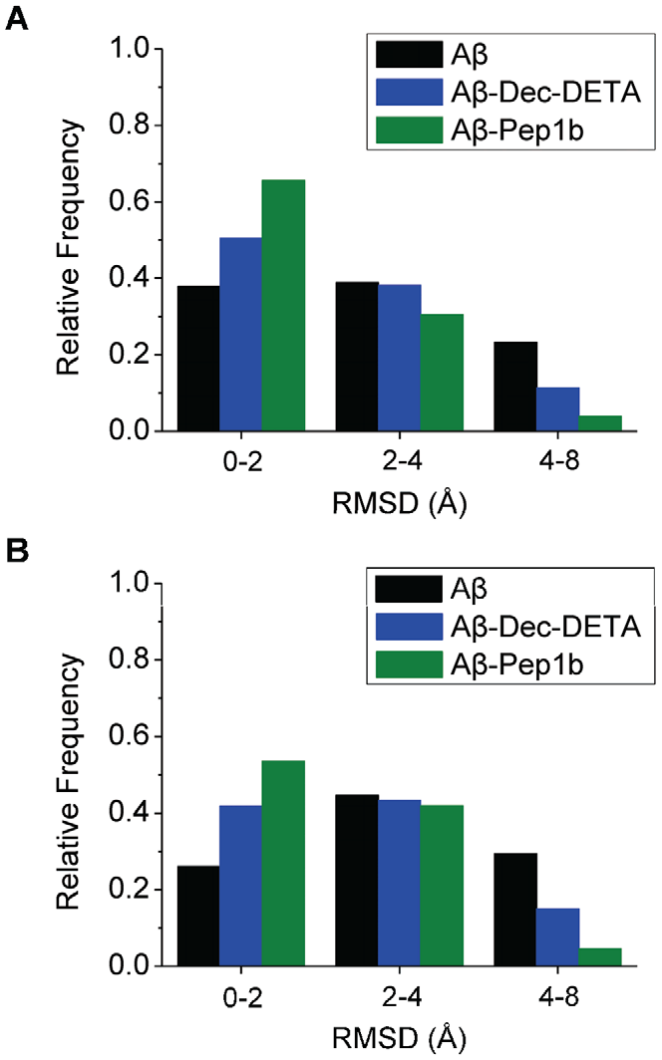
Histograms of RMSD of Aβ in the absence or presence of the ligands. The histograms of the Aβ (black bars), Aβ-Dec-DETA (blue bars), and Aβ-Pep1b (green bars) systems are shown. The histograms were obtained using the data of the whole simulations (A) and the second half of the simulations (B) of all ten trajectories of each system. The relative frequencies of the appearance of the Aβ structures sorted out by the three levels of RMSD (RMSD<2.0 Å, 2.0 Å≤RMSD<4.0 Å, and RMSD≥4.0 Å) of the Aβ middle region are indicated. The relative frequencies were calculated against total time of all ten trajectories of each system.

To examine behavior of the Aβ middle region during the simulations, the backbone RMSD during the whole simulations (Fig. 2A) and during the second half of the simulations (Fig. 2B) was calculated. By analyzing the backbone RMSD of the whole simulation of each trajectory, it was found that the Aβ helix was relatively stable during the first half of the simulations in five out of ten trajectories even if a ligand was not added to the system. For this reason, the second half of the simulations was used for this analysis. By visual inspection, it was determined that Aβ structures with small (RMSD<2.0 Å), medium (2.0 Å≤RMSD<4.0 Å), and large (RMSD≥4.0 Å) RMSD correspond to helical, moderately unwound, and highly unwound or elongated Aβ structures, respectively. Below we refer to these groups as peptide-conformation classes 1, 2, and 3, respectively. Both ligands, particularly Pep1b, increase the population of class 1 and decrease the population of class 3 (Fig. 2). During the second half of the simulations, the relative frequencies of class 1 and 3 in the presence of Dec-DETA are 1.6 and 0.5 times the frequencies for Aβ alone. In the presence of Pep1b the corresponding numbers are 2.1 and 0.2. Without a ligand class 3 is more populated than class 1 during the second half of the simulations, a situation which is reversed by both ligands (Fig. 2B).

**Figure 3:**
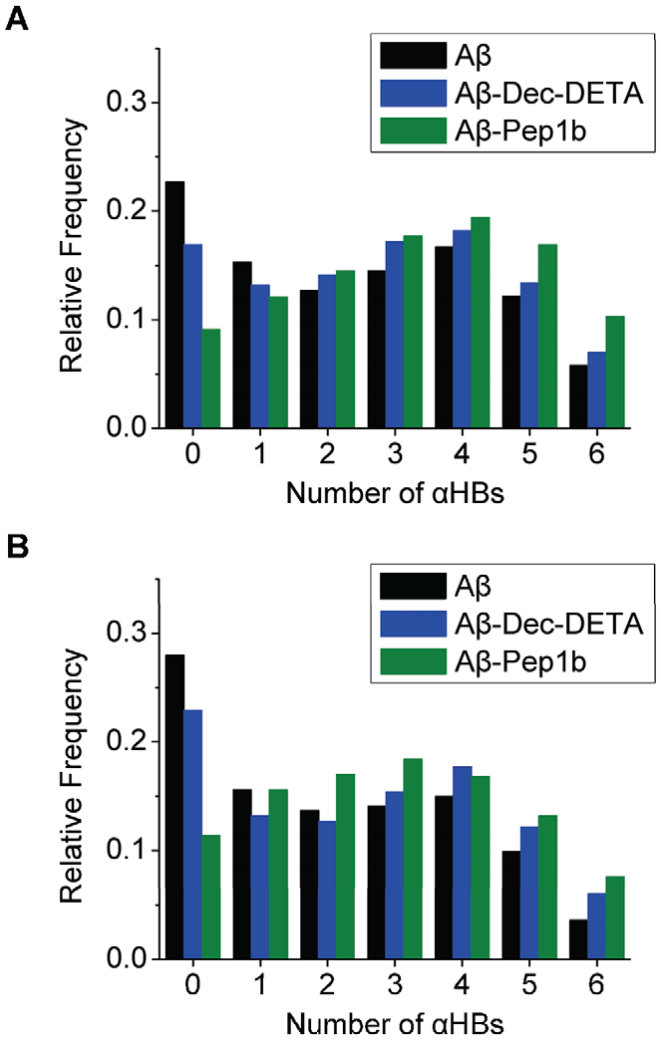
Histograms of the number of αHBs of Aβ in the absence or presence of the ligands. The histograms of the Aβ (black bars), Aβ-Dec-DETA (blue bars), and Aβ-Pep1b (green bars) systems are shown. The histograms were obtained using the data of the whole simulations (A) and the second half of the simulations (B) of all ten trajectories of each system. The relative frequencies of the appearance of the Aβ structures sorted out by the number of *n* αHBs (*n* = 0−6) of the Aβ middle region are indicated. The relative frequencies were calculated against total time of all ten trajectories of each system.

The number of αHBs in the helix was calculated to further characterize the behavior of the Aβ middle region (Fig. 3). The relative frequency of Aβ structures with no αHBs is decreased by addition of both ligands, particularly by addition of Pep1b (Fig. 3). This aspect is observed especially in the second half of the simulations (Fig. 3B). The existence of Aβ structures with five or six αHBs is increased by addition of both ligands, particularly by addition of Pep1b. During the second half of the simulations, the probability to find at least five αHBs is 1.3 and 1.5 times higher for Aβ in the presence of Dec-DETA and Pep1b, respectively, compared to Aβ alone.

These results indicate that both addition of Dec-DETA and Pep1b are effective in stabilizing the Aβ central helix and that Pep1b is somewhat more effective than Dec-DETA.

### Interactions between the Ligands and Aβ

**Figure 4:**
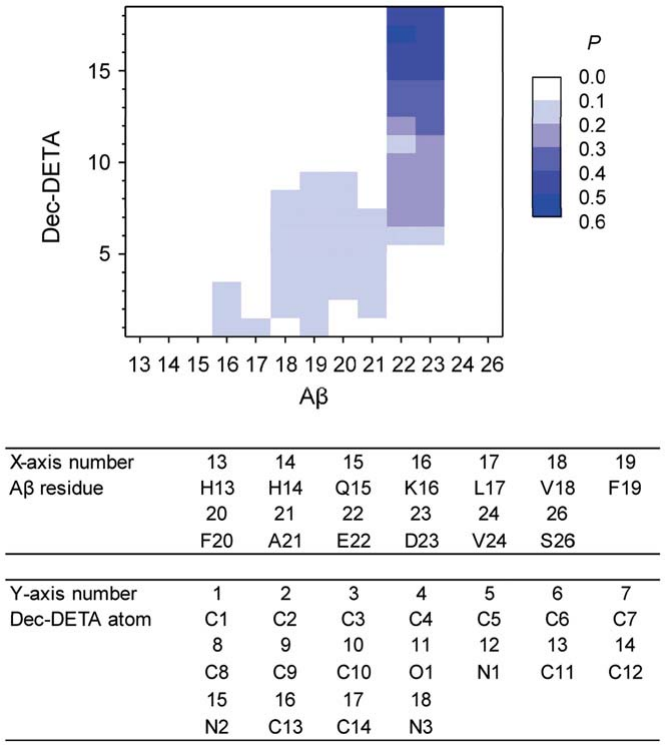
Contact map of the Aβ-Dec-DETA complex. The probability (0.0≤*P*<0.6) of the contact between the center of geometry of sidechain heavy atoms of each Aβ residue and each Dec-DETA heavy atom is colored (white to blue grids). The probability was calculated using the data obtained from the whole simulations of all ten trajectories. The Aβ residues and Dec-DETA atoms corresponding to the X and Y-axis numbers, respectively, are listed below the map.

**Figure 5:**
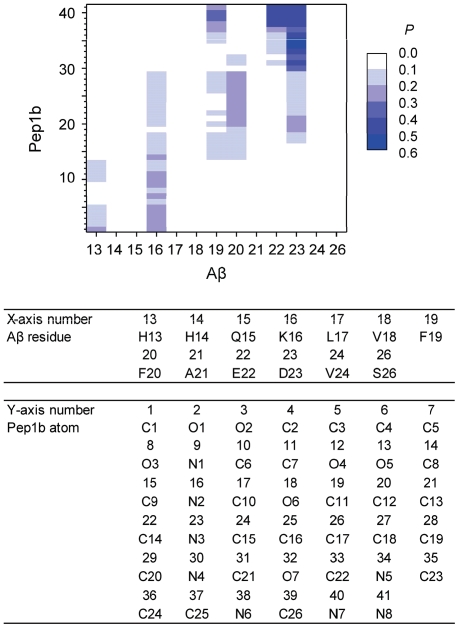
Contact map of the Aβ-Pep1b complex. The probability (0.0≤*P*<0.6) of the contact between the center of geometry of sidechain heavy atoms of each Aβ residue and each Pep1b heavy atom is colored (white to blue grids). The probability was calculated using the data obtained from the whole simulations of all ten trajectories. The Aβ residues and Pep1b atoms corresponding to the X and Y-axis numbers, respectively, are listed below the map.

To examine whether the ligands were in contact with Aβ as they were designed (Fig. 1), the contact maps (Fig. 4 and 5) were analyzed. All contact probabilities are lower than 0.6, indicating that the ligands sometimes detached from Aβ. By visual inspection of the trajectories, we found both Aβ and the ligands to be quite flexible and that the ligands sometimes detached from Aβ but bound to Aβ again. High contact probabilities (0.4≤P<0.6) are observed for contacts between the basic functional groups (N2 and N3) of Dec-DETA and the acidic residues (E22 and D23) of Aβ and for contacts between the basic functional groups (N5, N7, and N8) of Pep1b and the acidic residues (E22 and D23) of Aβ. Contacts between the acidic functional groups (O1, O2, O4, and O5) of Pep1b and the basic residues (H13 and K16) of Aβ occur with medium probabilities (0.2≤P<0.3). Contacts between the Dec-DETA hydrocarbon tail (C1–C9) and the Aβ middle nonpolar part are distributed from L17 to A21 of Aβ, although the probabilities are low (0.1≤P<0.2). In contrast, contacts between the Pep1b indole group (C13–C20 and N3) and the Aβ middle nonpolar part are localized at F19 and F20 of Aβ, with a preference for F20 (0.2≤P<0.3). Thus, the contact maps show that the ligands were in contact with Aβ as they were designed, even though the ligands sometimes detached from Aβ.

Contact maps from simulations of both Aβ-ligand complexes at 310 K (Fig. S1 and S2) show higher probabilities (P≥0.6) than at 360 K, and the distribution of contacts in each Aβ-ligand complex is more localized at 310 K than at 360 K. This is because the conformations of Aβ and the ligands did not change so much and the ligands almost always bound to Aβ at 310 K, in contrast to the motions of Aβ and the ligands at 360 K. However, the pattern of contacts in each Aβ-ligand complex at 310 K is similar to that at 360 K, and the main contacts of each Aβ-ligand complex at 310 K are almost the same as those at 360 K. Although motions of the ligands and Aβ are enhanced due to the increased temperature, interactions between the ligands and Aβ at the relatively high temperature are thus similar to those at the body temperature.

**Figure 6:**
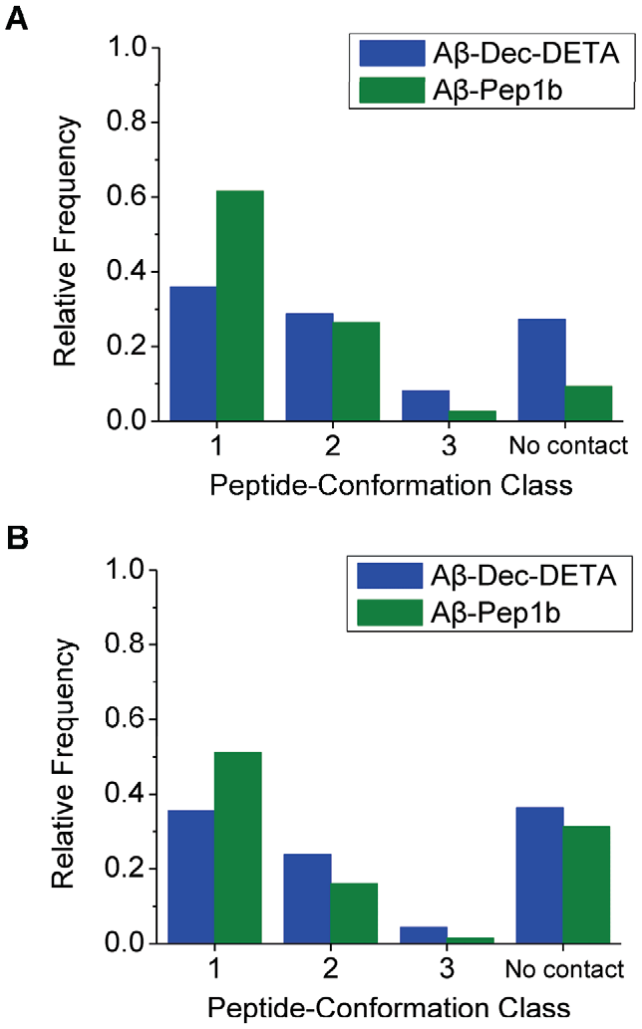
Histograms of polar and nonpolar Aβ-ligand contacts. The histograms of ligand contacts (Dec-DETA, blue bars; Pep1b, green bars) to the three peptide-conformation classes ((1) RMSD<2.0 Å, (2) 2.0 Å≤RMSD<4.0 Å, and (3) RMSD≥4.0 Å) were obtained using the data of the whole simulations of all ten trajectories of each system. In calculations of relative frequencies, the occurrence of the polar or nonpolar contacts for each peptide-conformation class was divided by total time of all ten trajectories of each system. (A) Relative frequencies of the polar contacts with at least one Aβ-ligand HB. (B) Relative frequencies the nonpolar contacts with at least one Aβ-ligand C-C or C-N contact. The contacts between the Aβ middle nonpolar part (residues 17–21) and the hydrocarbon tail of Dec-DETA (C1–C9) or the indole group of Pep1b (C13–C20 and N3) were used for this analysis.

To understand polar interactions between the ligands and Aβ, the existence of HBs between the ligands and Aβ was analyzed for the three peptide-conformation classes; the frequency of time when the ligands do not form any HBs with Aβ regardless of the peptide conformation was also calculated (Fig. 6A). In total, Dec-DETA and Pep1b form at least one HB with Aβ for 73% and 91% of the total time, respectively (Fig. 6A). When we consider only the helical class 1 conformations, Pep1b is in polar contact (hydrogen bonding contact) with Aβ 1.7 times as often as Dec-DETA (Fig. 6A). The fraction of the occurrence of the polar contacts for each peptide-conformation class (Table 2) shows that Pep1b binds to the Aβ structures in class 1 with higher probability than to the Aβ structures in classes 2 and 3, whereas Dec-DETA binds to all three peptide-conformation classes with similar probabilities. Besides, the fraction of the occurrence of the polar contacts for the class 1 conformations is higher for Pep1b than for Dec-DETA (Table 2). These data indicate that Pep1b binds more specifically to helical Aβ than Dec-DETA does. Additionally, the Aβ structures in class 1 form one more HB on average with Pep1b than with Dec-DETA (Table 3).

**Table 2:** Fractions of polar and nonpolar contacts between Aβ and Dec-DETA or Pep1b for each peptide-conformation class^a^.

	polar contacts^b^			nonpolar contacts^c^		
ligand	class 1	class 2	class 3	class 1	class 2	class 3
Dec-DETA	0.71	0.75	0.71	0.70	0.62	0.38
Pep1b	0.94	0.87	0.67	0.78	0.53	0.36

aThe fractions were calculated for the three peptide-conformation classes ((1) RMSD<2.0 Å, (2) 2.0 Å≤RMSD<4.0 Å, and (3) RMSD≥4.0 Å) using all ten trajectories of each system. The occurrence of the polar or nonpolar contacts for each peptide-conformation class was divided by the frequency of each peptide-conformation class.

bThe polar contacts were determined by the existence of at least one HB between Aβ and Dec-DETA or Pep1b.

cThe nonpolar contacts were determined by the existence of at least one C-C or C-N contact between the Aβ middle nonpolar part and the nonpolar part of Dec-DETA or Pep1b.

**Table 3:** Average number of polar and nonpolar contacts between Aβ and Dec-DETA or Pep1b for each peptide-conformation class^a^.

	polar contacts^b^			nonpolar contacts^c^		
ligand	class 1	class 2	class 3	class 1	class 2	class 3
Dec-DETA	2.8±1.2	2.9±1.3	3.0±1.3	17.3±10.0	16.7±10.0	11.4±9.0
Pep1b	3.8±1.7	3.7±1.6	3.4±1.7	18.7±12.7	15.3±12.3	14.1±10.1

aThe mean values (± standard deviations) were calculated for the three peptide-conformation classes ((1) RMSD<2.0 Å, (2) 2.0 Å≤RMSD<4.0 Å, and (3) RMSD≥4.0 Å) using all ten trajectories of each system.

bAveraged over periods with at least one HB between Aβ and Dec-DETA or Pep1b.

cAveraged over periods with at least one C-C or C-N contact between the Aβ middle nonpolar part and the nonpolar part of Dec-DETA or Pep1b.

In a similar way, we analyzed the existence of nonpolar interactions (C-C and C-N contacts) between the nonpolar groups of the ligands (the hydrocarbon tail of Dec-DETA and the indole group of Pep1b) and the middle nonpolar part (residues 17–21) of Aβ for the three peptide-conformation classes; the frequency of time when the ligands do not have any C-C and C-N contacts with Aβ regardless of the peptide conformation was also calculated (Fig. 6B). In total, the nonpolar groups of Dec-DETA and Pep1b have at least one C-C or C-N contact with the middle nonpolar part of Aβ for 64% and 69% of the total time, respectively (Fig. 6B). When we consider only the class 1 conformations, Pep1b is in nonpolar contact with Aβ 1.4 times as often as Dec-DETA (Fig. 6B). The fraction of the occurrence of the nonpolar contacts for the class 1 conformations is higher for Pep1b than for Dec-DETA (Table 2). These data indicate that the indole group of Pep1b has contacts with the middle nonpolar part of helical Aβ more frequently than the hydrocarbon tail of Dec-DETA does. Additionally, the Aβ structures in class 1 have one more C-C or C-N contact on average with Pep1b than with Dec-DETA (Table 3).

**Figure 7:**
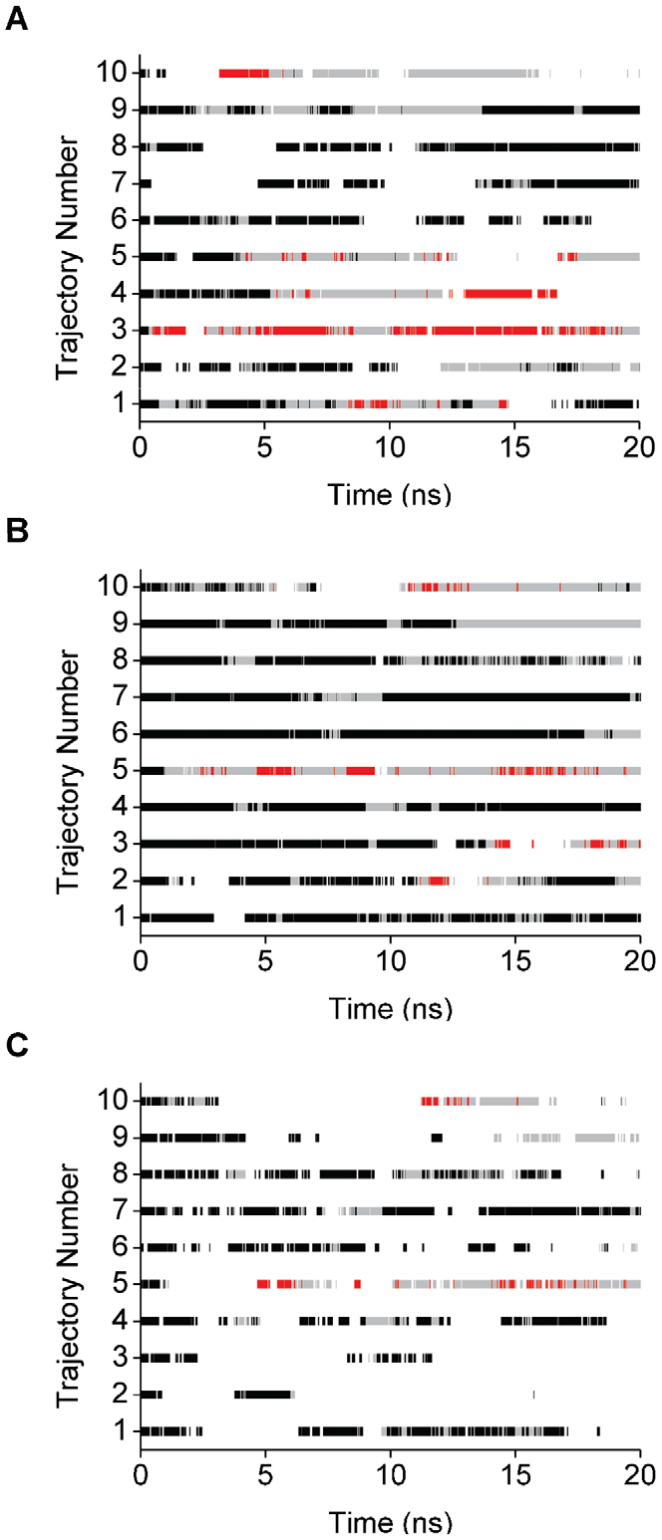
Timelines of Aβ-ligand contacts. Timelines showing the presence of at least one Aβ-ligand hydrogen bonding contact for Aβ-Dec-DETA (A) and Aβ-Pep1b (B), and for Aβ-Pep1b (C) also when both kinds of HBs between Aβ and Pep1b (between the Aβ acidic residue sidechains and the Pep1b basic functional groups, and between the Aβ basic residue sidechains and the Pep1b acidic functional groups) were formed at the same time. The ligand-binding events are distinguished by using different colors for the three peptide-conformation classes 1 (black bars), 2 (gray bars), and 3 (red bars).

To further understand interactions between the ligands and Aβ, we also anlyzed the existence of HBs between the ligands and Aβ for the three peptide-conformation classes in each individual trajectory (Fig. 7). The intermittent lines for the Aβ-Dec-DETA (Fig. 7A) and Aβ-Pep1b (Fig. 7B) complexes show that both ligands sometimes detach from Aβ and bind again to Aβ. Long durations of the ligands in hydrogen bonding contact with the class 1 conformations are more frequent for Aβ-Pep1b (Fig. 7B) than for Aβ-Dec-DETA (Fig. 7A). This shows that, compared to Dec-DETA, Pep1b binds to the helical conformations of Aβ more constantly and is thus more effective in stabilizing the Aβ central helix. In contrast, long durations of the ligands in hydrogen bonding contact with the class 3 conformations are more frequent for Aβ-Dec-DETA than for Aβ-Pep1b, indicating that Dec-DETA binds to the highly unwound or elongated conformations of Aβ for longer periods than Pep1b.

In addition, to examine whether Pep1b binds to Aβ with both acidic and basic functional groups at the same time during the simulation, we analyzed events when both basic and acidic functional groups of Pep1b form HBs with the sidechains of the acidic and basic residues of Aβ, respectively, at the same time (Fig. 7C). All trajectories begin with Aβ in conformation class 1 and Pep1b bound with both acidic and basic groups, and in five trajectories (1, 4, 6, 7, and 8), this is also observed frequently for class 1 during the whole simulation, indicating that Pep1b can bind to helical Aβ with both acidic and basic functional groups at the same time from the beginning to the end of the simulation. In three of these trajectories (1, 4, and 7), Aβ maintained its helical conformation and had not unfolded by the end of the simulation (Table 1, group A).

As mentioned above, the group A trajectories exhibit non-unfolding or refolding of Aβ. In one of the group A trajectories of each complex, trajectory 1 of the Aβ-Dec-DETA complex and trajectory 2 of the Aβ-Pep1b complex, Aβ refolded to a helical conformation after being highly unwound during part of the simulations (Fig. 7A and 7B). Dec-DETA was bound to Aβ during the first partial unfolding (8–11 ns) and refolding (11–12 ns) events, and during the first half of the second partial unfolding event (13–16 ns) but not during the second refolding event (16–17 ns) in trajectory Aβ-Dec-DETA-1 (Fig. 7A). Pep1b was bound to Aβ during the partial unfolding event (11–15 ns) except for a short break (12.5–13.5 ns), and was bound to Aβ during the refolding event (15–16 ns) in trajectory Aβ-Pep1b-2 (Fig. 7B). By visual inspection, we found that the charged functional groups of both Dec-DETA and Pep1b formed constant polar contacts with the charged sidechains of Aβ when the ligands were bound to Aβ during the partial unfolding and refolding periods, whereas the nonpolar contacts were intermittent.

According to our previous study [17], the Aβ central helix does not completely unfold in cases where any of the three steps of the three-step mechanism, which was proposed for the complete unfolding of the Aβ central helix, is missing: 1) sufficient loss of α-helical backbone hydrogen bonds, 2) strong interactions between nonpolar sidechains, and 3) strong interactions between polar sidechains. Here we observed that Aβ did not completely unfold due to the lack of steps 3 and 2 in the first and second partial unfolding events, respectively, in trajectory Aβ-Dec-DETA-1, and due to the lack of step 3 in the partial unfolding event in trajectory Aβ-Pep1b-2.

These data suggest that strong inter-molecular interactions between the ligand polar groups and the Aβ polar sidechains prevent intra-molecular interactions between the Aβ polar sidechains, thus blocking the third step of the unfolding mechanism in trajectories Aβ-Dec-DETA-1 and Aβ-Pep1b-2. In this way Aβ is inhibited from complete unfolding and instead Aβ refolding is facilitated.

### Ligand-Binding to Unwound Aβ

As shown above, both ligands were able to bind to the unwound Aβ structures in the peptide-conformation class 3, and long durations of the ligand-binding for class 3 were more frequent for the Aβ-Dec-DETA complex than for the Aβ-Pep1b complex (Fig. 7). This result suggests that both ligands, particularly Dec-DETA, have the possibility of being involved in the polymerization which occurs after the unfolding of the Aβ central helix. To examine how the ligands interact with unwound Aβ, we analyzed the ligand-binding events for class 3 in each individual trajectory in detail. Details of two Aβ-Dec-DETA trajectories and one Aβ-Pep1b trajectory, which exhibit long durations of the ligand-binding for class 3, are described below. Note that similar features were observed in the other trajectories of each Aβ-ligand simulation.

**Figure 8:**
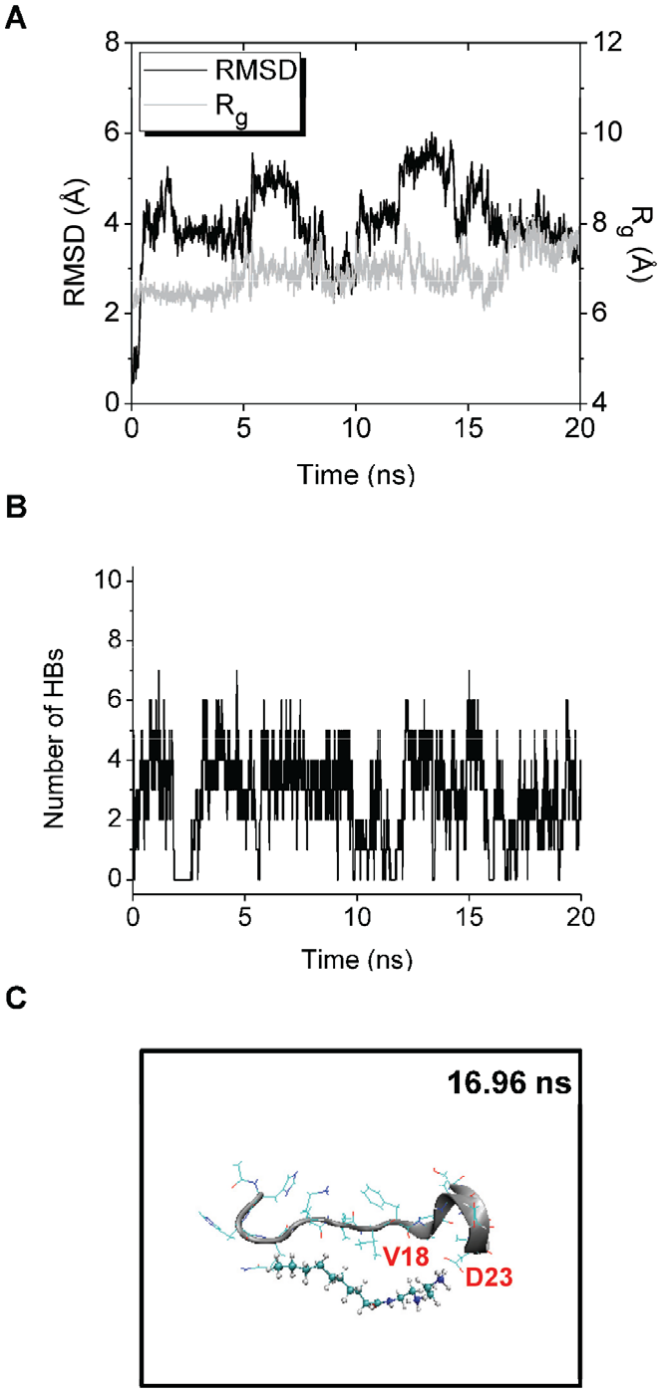
Structural changes of trajectory 3 of the Aβ-Dec-DETA system. The RMSD and R_g_ of the Aβ middle region (A) and the number of HBs between Aβ and Dec-DETA (B) are shown. The structure obtained at 16.96 ns (with large RMSD (4.12 Å), large R_g_ (8.03 Å), and two HBs) is also shown (C).

In trajectory 3 of the Aβ-Dec-DETA simulation, R_g_ of Aβ reaches a peak (R_g_≥7.5 Å) at around 17 ns (Fig. 8A), and one or two HBs between Dec-DETA and Aβ are formed at the time (Fig. 8B). At around the time of the R_g_ peak, β-strand-like forms of Aβ bound by Dec-DETA were observed, and a typical structure of these forms was obtained at 16.96 ns (Fig. 8C). In this structure, two HBs are formed between Aβ and Dec-DETA (Table 4), and the hydrocarbon sidechains of Aβ are located close to the hydrocarbon chain of Dec-DETA (The Cγ1(V18)-C5(Dec-DETA), Cγ1(V18)-C6(Dec-DETA), and Cγ2(V18)-C8(Dec-DETA) distances are 3.97, 3.93, and 4.00 Å, respectively.).

**Table 4:** HBs formed between Aβ and Dec-DETA or Pep1b at the specific time.

				Aβ				ligand
complex	trajectory	time (ns)	number of HBs	residue	(location)	atom		atom
Aβ-Dec-DETA	3	16.96	2	D23	(sidechain)	Oδ1	—	HN3
				D23	(sidechain)	Oδ2	—	HN3
	4	14.29	4	A21	(backbone)	HN	—	O1
				E22	(sidechain)	Oε1	—	HN2
				E22	(sidechain)	Oε1	—	HN3
				E22	(sidechain)	Oε2	—	HN3
Aβ-Pep1b	5	5.15	7	H13	(backbone)	HN	—	O4
				H13	(sidechain)	HNδ1	—	O4
				H13	(sidechain)	HNδ1	—	O5
				E22	(sidechain)	Oε2	—	HN7
				E22	(sidechain)	Oε2	—	HN8
				D23	(sidechain)	Oδ1	—	HN6
				D23	(sidechain)	Oδ1	—	HN8
		6.36	6	H13	(sidechain)	HNδ1	—	O5
				H14	(sidechain)	HNδ1	—	O3
				E22	(sidechain)	Oε2	—	HN8
				D23	(sidechain)	Oδ1	—	HN5
				D23	(sidechain)	Oδ2	—	HN6
				D23	(sidechain)	Oδ2	—	HN8
		9.12	2	E22	(sidechain)	Oε1	—	HN7
				E22	(sidechain)	Oε2	—	HN8
		10.47	4	H13	(backbone)	HN	—	O4
				H13	(sidechain)	HNδ1	—	O5
				E22	(sidechain)	Oε1	—	HN6
				E22	(sidechain)	Oε2	—	HN8

**Figure 9:**
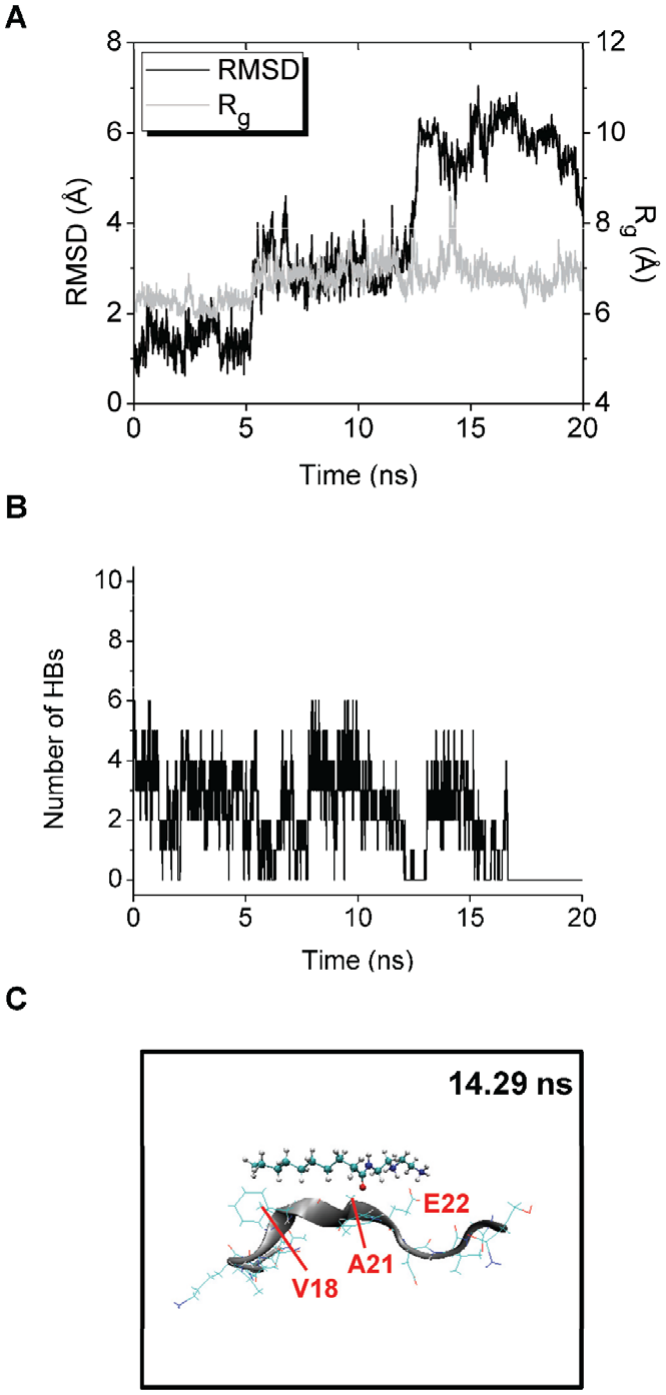
Structural changes of trajectory 4 of the Aβ-Dec-DETA system. The RMSD and R_g_ of the Aβ middle region (A) and the number of HBs between Aβ and Dec-DETA (B) are shown. The structure obtained at 14.29 ns (with large RMSD (4.59 Å), large R_g_ (8.48 Å), and four HBs) is also shown (C).

In trajectory 4 of the Aβ-Dec-DETA simulation, R_g_ of Aβ reaches a peak at around 14 ns (Fig. 9A), and at least two HBs between Dec-DETA and Aβ are formed at the time (Fig. 9B). β-strand-like forms of Aβ bound by Dec-DETA were observed at around the time of the R_g_ peak, and a typical structure of these forms was obtained at 14.29 ns (Fig. 9C). In this structure, four HBs are formed between Aβ and Dec-DETA (Table 4), and the hydrocarbon sidechains of Aβ are located close to the hydrocarbon chain of Dec-DETA (The Cγ2(V18)-C2(Dec-DETA), Cγ2(V18)-C3(Dec-DETA), and Cβ(A21)-C11(Dec-DETA) distances are 4.00, 4.00, and 4.13 Å, respectively.).

In both Aβ-Dec-DETA structures obtained at the times of the R_g_ peaks in trajectories 3 and 4, the hydrocarbon chain of Dec-DETA is located along the backbone of β-strand-like Aβ, and thus, β-strand-like Aβ and Dec-DETA form parallel conformations (Fig. 8C and 9C). The parallel conformations of the Aβ-Dec-DETA complex can be formed, due to the non-bulky conformation of Dec-DETA.

**Figure 10:**
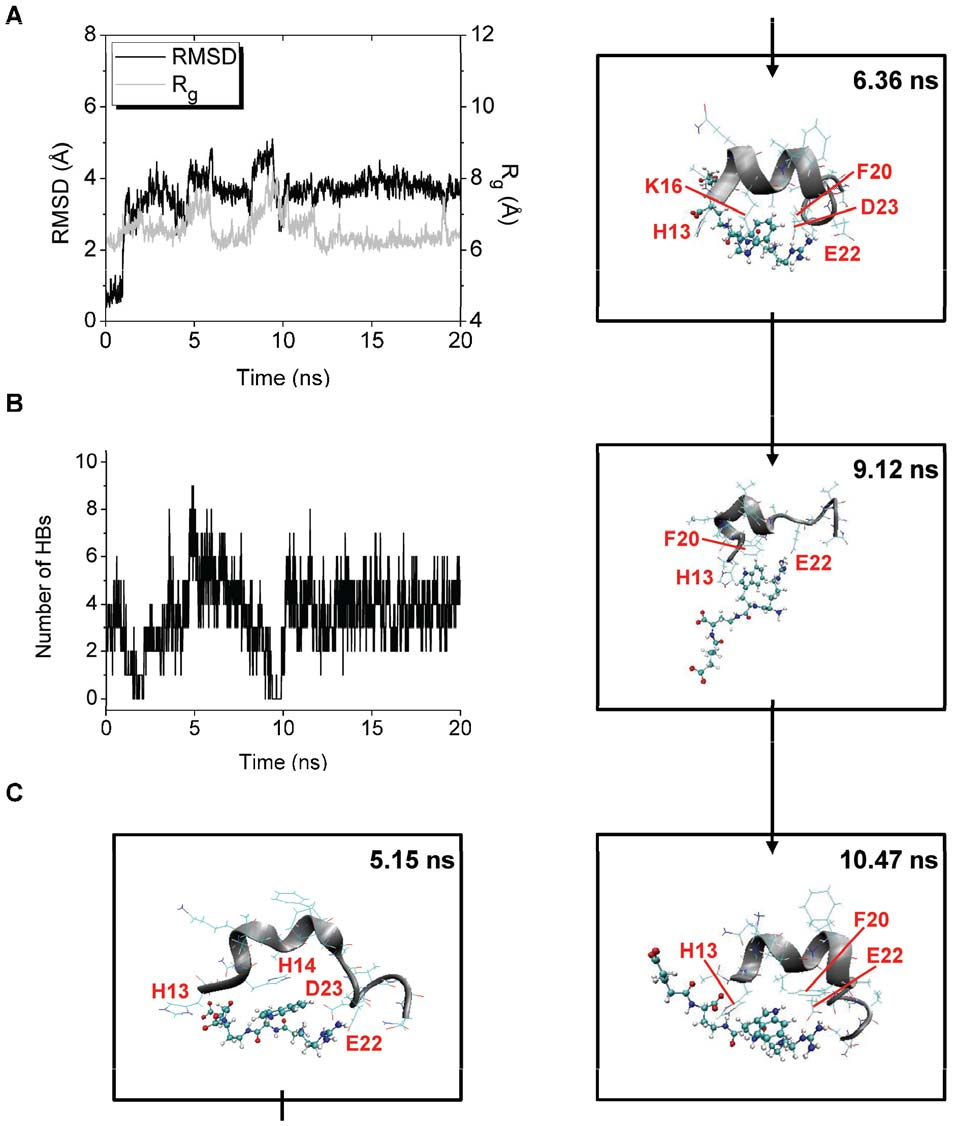
Structural changes of trajectory 5 of the Aβ-Pep1b system. The RMSD and R_g_ of the Aβ middle region (A) and the number of HBs between Aβ and Pep1b (B) are shown. The structures obtained at 5.15 ns (with large RMSD (4.10 Å), large R_g_ (7.56 Å), and seven HBs), at 6.36 ns (with medium RMSD (3.64 Å), small R_g_ (6.16 Å), and six HBs), at 9.12 ns (with large RMSD (4.36 Å), large R_g_ (7.97 Å), and two HBs), and at 10.47 ns (with medium RMSD (3.45 Å), small R_g_ (6.56 Å), and four HBs) are also shown (C).

In trajectory 5 of the Aβ-Pep1b simulation, R_g_ of Aβ reaches peaks at around 5 and 9 ns (Fig. 10A). The number of HBs between Pep1b and Aβ is more than four at around 5 ns and is less than four at around 9 ns (Fig. 10B). These data show that Pep1b is tightly bound to the highly unwound or elongated Aβ at around the time of the first R_g_ peak but not at around the time of the second R_g_ peak. After the first and second R_g_ peaks, decreases in R_g_ are observed together with decreases in RMSD (Fig. 10A), and several HBs between Pep1b and Aβ are formed at these times (Fig. 10B), showing that Pep1b is bound to Aβ which adopts compact forms at these times.

β-strand-like forms of Aβ bound by Pep1b were not observed at around the times of both R_g_ peaks, and instead, bent forms of Aβ bound by Pep1b were observed. Typical structures of these forms observed at around the times of the first and second R_g_ peaks were obtained at 5.15 and 9.12 ns, respectively (Fig. 10C). After the times of the first and second R_g_ peaks, compact and partially helical forms of Aβ bound by Pep1b were observed, and typical structures of these forms were obtained at 6.36 and 10.47 ns (Fig. 10C).

At the time of the first R_g_ peak (5.15 ns), seven HBs are formed between Aβ and Pep1b (Table 4), and the H14 imidazole ring of Aβ is located close to the indole ring of Pep1b (The Cδ2(H14)-C15(Pep1b) and Cδ2(H14)-C20(Pep1b) distances are 3.27 and 3.42 Å, respectively.). The backbone of Aβ is bent by the electrostatic interactions and by the auxiliary van der Waals interactions (Fig. 10C). After the time of the first R_g_ peak (6.36 ns), six HBs are formed between Aβ and Pep1b (Table 4), and the K16 sidechain and the F20 benzene ring of Aβ are located close to the indole ring of Pep1b (The Cγ(K16)-C19(Pep1b) and Cγ(F20)-C19(Pep1b) distances are 3.69 and 3.93 Å, respectively.). The helical form of the backbone of Aβ is partially (Q15-A21) reconstructed by the electrostatic interactions and by the auxiliary van der Waals interactions (Fig. 10C). At the time of the second R_g_ peak (9.12 ns), two HBs are formed between Aβ and Pep1b (Table 4), and the H13 imidazole ring and the F20 benzene ring of Aβ are located close to the indole ring of Pep1b (The Cδ2(H13)-C16(Pep1b) and Cγ(F20)-C20(Pep1b) distances are 3.63 and 3.66 Å, respectively.). The backbone of Aβ is partially (H13-F20) bent by the van der Waals interactions, though the backbone of Aβ is partially (A21-S26) elongated (Fig. 10C). After the time of the second R_g_ peak (10.47 ns), four HBs are formed between Aβ and Pep1b (Table 4), and the H13 imidazole ring and the F20 benzene ring of Aβ are located close to the indole ring of Pep1b (The Cγ(H13)-C17(Pep1b) and Cε1(F20)-C20(Pep1b) distances are 3.82 and 3.34 Å, respectively.). The helical form of the backbone of Aβ is partially (Q15-A21) reconstructed by the electrostatic interactions and by the auxiliary van der Waals interactions (Fig. 10C).

As shown in the Aβ-Pep1b structures obtained in trajectory 5, the basic and acidic functional groups of Pep1b can simultaneously interact with the sidechains of the acidic and basic residues of Aβ, respectively. In addition, the aromatic ring of Pep1b can at the same time interact with the aromatic rings of Aβ. Aβ therefore cannot easily convert to a β-strand-like form because of these electrostatic and van der Waals interactions. Even if Aβ would be assumed to be a β-strand-like form, parallel conformations of the Aβ-Pep1b complex cannot be formed, due to the bulky conformation of Pep1b.

## Discussion

The effects of the two ligands (Dec-DETA and Pep1b) on the stability of the Aβ central helix (residues 15–24) were investigated by using MD simulations. Detailed information on structural changes upon loss of helicity in the presence of the ligands was also examined, which might explain the observed difference in structures of Aβ fibrils in the presence of Dec-DETA or Pep1b.

As indicated mainly by the Aβ backbone RMSD *vs* the initial structure and by the existence of αHBs of Aβ, the Aβ central helix completely unfolded by the end of the simulation in three out of ten trajectories in the absence of a ligand, whereas it completely unfolded in only one out of ten trajectories in the presence of Dec-DETA and did not completely unfold in any of ten trajectories in the presence of Pep1b. Compared to Aβ alone, the probability of the Aβ helical state (more than 2/3 of all the αHBs are formed) during the second half of the simulations is 1.3 and 1.5 times higher for Aβ in the presence of Dec-DETA and Pep1b, respectively. It was thus indicated that the stability of the Aβ central helix was increased by both ligands, in agreement with the experimental data [12]. It was also indicated that the ability of Pep1b to stabilize the Aβ central helix is higher than that of Dec-DETA, which was not shown in the previous experimental study [12].

The analysis of the ligand-binding events clearly showed that Pep1b binds to the Aβ central helix longer time than Dec-DETA does. A main reason for this is that Pep1b has both basic and acidic functional groups which can simultaneously bind to the acidic and basic residues of Aβ, respectively, whereas Dec-DETA has only the basic functional groups. The inter-molecular interactions between the Aβ polar residues and the ligand polar functional groups are important in stabilizing the Aβ central helix, because they can prevent intra-molecular interactions between the Aβ polar residues that induce complete unfolding of the Aβ central helix [17]. An additional reason would be that Pep1b includes a centrally placed aromatic ring which straddles the Aβ middle nonpolar part (residues 17–21) when the basic and acidic functional groups of Pep1b simultaneously bind to the acidic and basic residues of Aβ, respectively. The inter-molecular interactions between the Aβ middle nonpolar part and the ligand nonpolar part are likely to be important in stabilizing the Aβ central helix, since the Aβ middle nonpolar part includes the three nonpolar residues (VFF) that have low α-helical propensities and high β-strand propensities [39], [40].

This analysis also showed that both ligands can bind to highly unwound or elongated forms of Aβ. Dec-DETA was found to be able to form parallel conformations with β-strand-like forms of Aβ. In contrast, Pep1b was found not to be able to form parallel conformations with β-strand-like Aβ, due to the bulky conformation of Pep1b, and instead, Pep1b was found to bend unwound Aβ by the charge-charge interactions and by interactions between the aromatic rings. Therefore, it may be suggested that Dec-DETA could be included upon formation and extension of β-sheets to Aβ fibrils while being sandwiched between the two β-strands (residues 18–26 and 31–42) or being associated with the surface of a β-sheet, thus giving rise to fibrils with an alternative structure. On the other hand, Pep1b bound to unwound Aβ may disturb the extension of β-sheets.

To summarize, it appears that Pep1b is somewhat more effective in stabilizing the Aβ central helix than Dec-DETA. In addition, the difference in conformations between the unwound-Aβ complexes bound by Dec-DETA and by Pep1b could be a reason why Aβ incubated with Dec-DETA and with Pep1b form thicker-than-normal and shorter-than-normal fibrils, respectively, as reported by the previous experimental study [12], though the physical and physiological consequence of Dec-DETA containing alternative fibrils in vitro and in vivo is unknown. Hence, our study indicates that, compared to Dec-DETA-like ligands, Pep1b-like ligands, which are capable of having charge-charge interactions with both the acidic and basic residues of the Aβ middle region, additional hydrophobic interactions with the Aβ middle nonpolar part, and bulky conformations, appear to be more effective in inhibiting unwinding of helical Aβ and also in preventing subsequent association of unwound Aβ.

## Supporting Information

Figure S1 **Contact map of the Aβ-Dec-DETA complex at 310 K.** The probability (0.0≤*P*<1.0) of the contact between the center of geometry of sidechain heavy atoms of each Aβ residue and each Dec-DETA heavy atom is colored (white to blue grids). The probability was calculated using the data obtained from the whole simulation of one trajectory. The Aβ residues and Dec-DETA atoms corresponding to the X and Y-axis numbers, respectively, are listed below the map.
(TIFF)

Figure S2 **Contact map of the Aβ-Pep1b complex at 310 K.** The probability (0.0≤*P*<1.0) of the contact between the center of geometry of sidechain heavy atoms of each Aβ residue and each Pep1b heavy atom is colored (white to blue grids). The probability was calculated using the data obtained from the whole simulation of one trajectory. The Aβ residues and Pep1b atoms corresponding to the X and Y-axis numbers, respectively, are listed below the map.
(TIFF)

Table S1 CHARMM force field parameters for the ligands.
(DOC)
