# Supplementary figures and images for: Effects of Ligands on Unfolding of the Amyloid β-Peptide Central Helix: Mechanistic Insights from Molecular Dynamics Simulations
Source: PLoS One. 2012 Jan 23;7(1):e30510. doi: 10.1371/journal.pone.0030510 (PMC3264620; doi:10.1371/journal.pone.0030510)

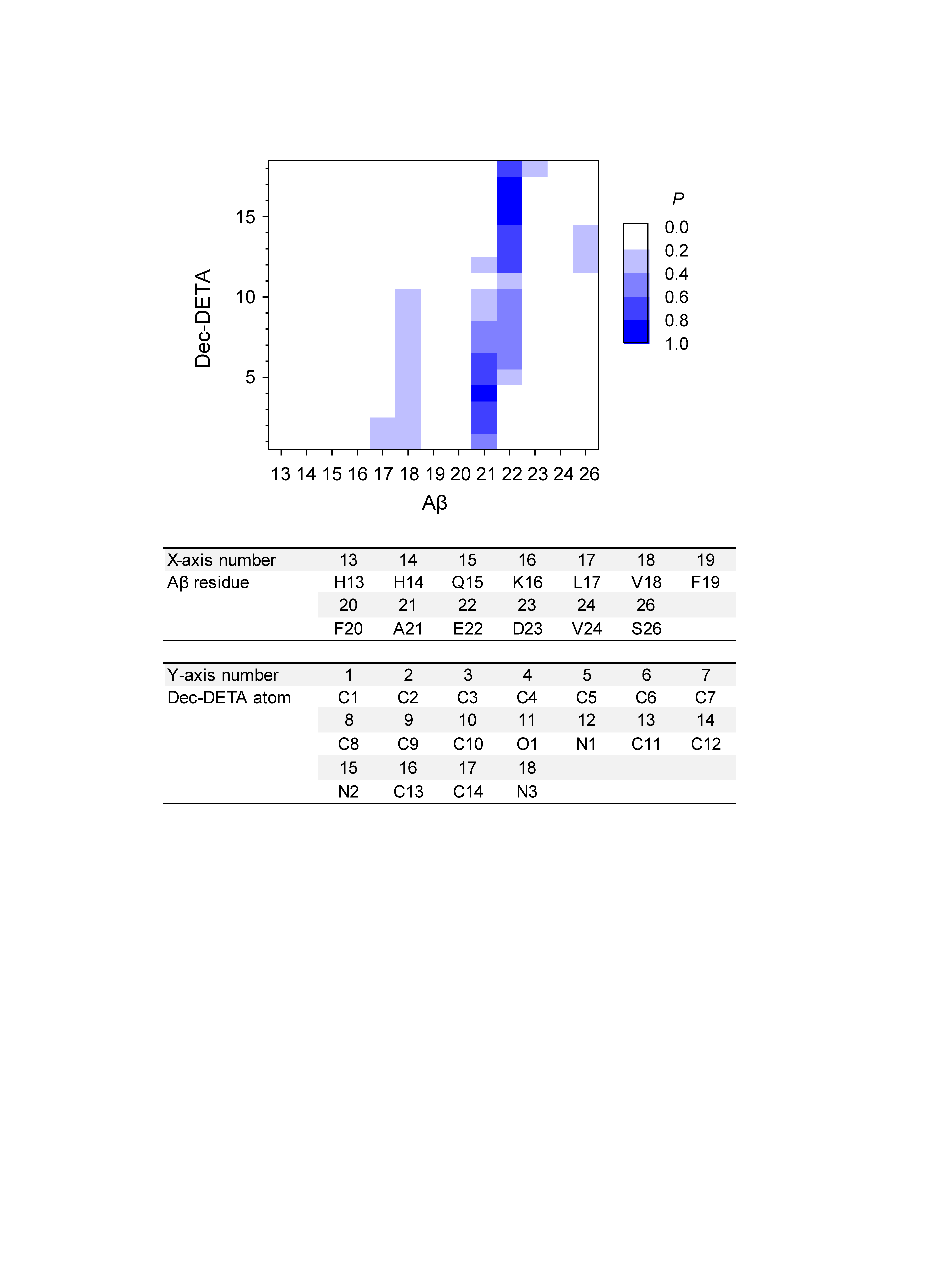

Supplement: Figure S1 — Contact map of the Aβ-Dec-DETA complex at 310 K. The probability (0.0≤P<1.0) of the contact between the center of geometry of sidechain heavy atoms of each Aβ residue and each Dec-DETA heavy atom is colored (white to blue grids). The probability was calculated using the data obtained from the whole simulation of one trajectory. The Aβ residues and Dec-DETA atoms corresponding to the X and Y-axis numbers, respectively, are listed below the map. (TIFF) [file pone.0030510.s001.tif]

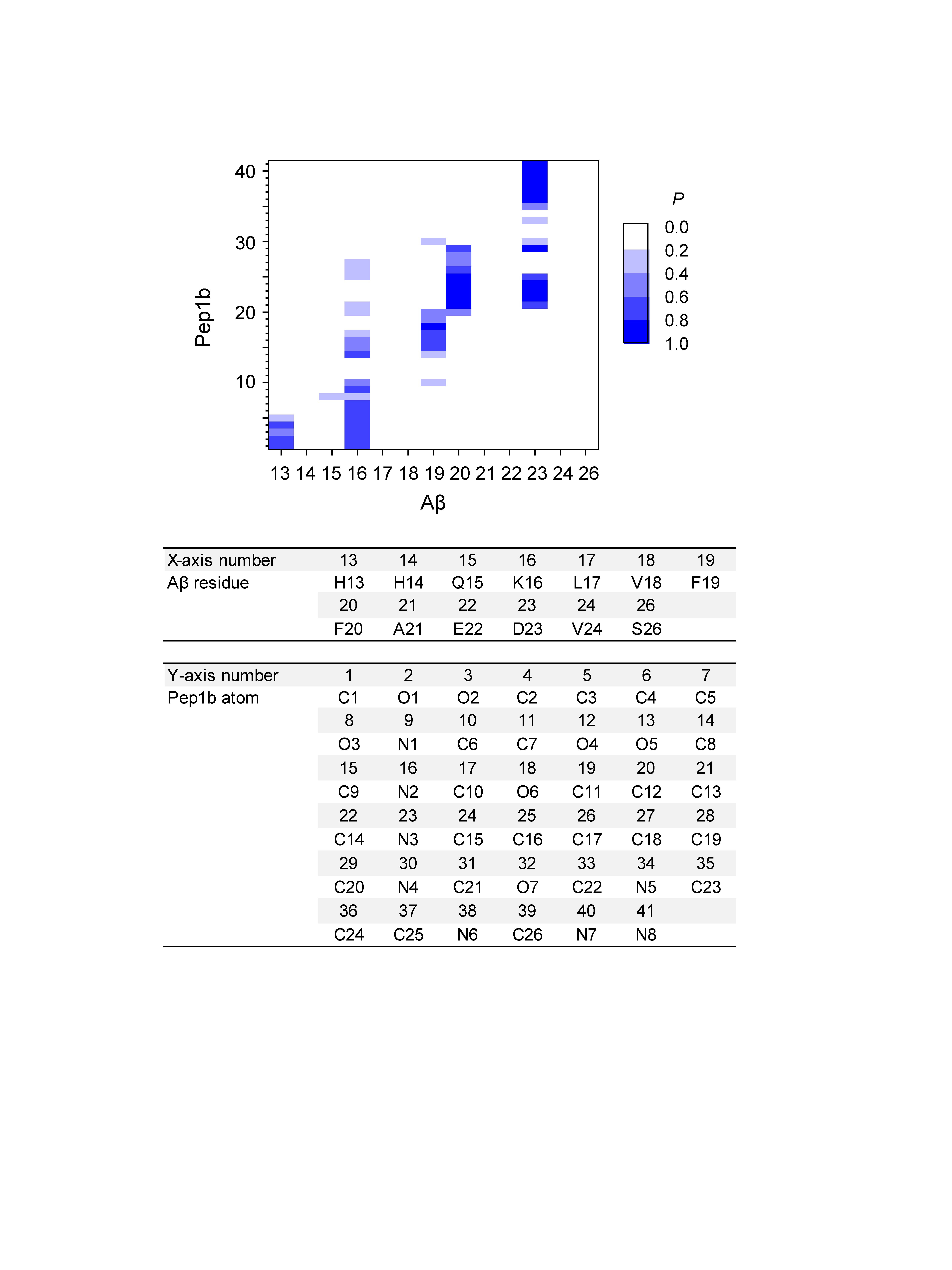

Supplement: Figure S2 — Contact map of the Aβ-Pep1b complex at 310 K. The probability (0.0≤P<1.0) of the contact between the center of geometry of sidechain heavy atoms of each Aβ residue and each Pep1b heavy atom is colored (white to blue grids). The probability was calculated using the data obtained from the whole simulation of one trajectory. The Aβ residues and Pep1b atoms corresponding to the X and Y-axis numbers, respectively, are listed below the map. (TIFF) [file pone.0030510.s002.tif]
