# Supplementary material for: Effects of Ligands on Unfolding of the Amyloid β-Peptide Central Helix: Mechanistic Insights from Molecular Dynamics Simulations
Source: PLoS One. 2012 Jan 23;7(1):e30510. doi: 10.1371/journal.pone.0030510 (PMC3264620; doi:10.1371/journal.pone.0030510)
Supplement: Table S1 — CHARMM force field parameters for the ligands. (DOC) [file pone.0030510.s003.doc]

**Table S1.** CHARMM force field parameters for the ligands.

**A.** Added CHARMM parameters for Dec-DETA.

Atom Atom Atom Ktheta (kcal/mole/rad**2) Theta0 (degree)

CT2 NH3 CT2 30.0 109.5

**B.** CHARMM topology for Dec-DETA.

Name Type Charge (e)

RESI 　　DECD 　2.00

GROUP

ATOM C1 CT3 -0.27

ATOM H11 HA 0.09

ATOM H12 HA 0.09

ATOM H13 HA 0.09

GROUP

ATOM C2 CT2 -0.18

ATOM H21 HA 0.09

ATOM H22 HA 0.09

GROUP

ATOM C3 CT2 -0.18

ATOM H31 HA 0.09

ATOM H32 HA 0.09

GROUP

ATOM C4 CT2 -0.18

ATOM H41 HA 0.09

ATOM H42 HA 0.09

GROUP

ATOM C5 CT2 -0.18

ATOM H51 HA 0.09

ATOM H52 HA 0.09

GROUP

ATOM C6 CT2 -0.18

ATOM H61 HA 0.09

ATOM H62 HA 0.09

GROUP

ATOM C7 CT2 -0.18

ATOM H71 HA 0.09

ATOM H72 HA 0.09

GROUP

ATOM C8 CT2 -0.18

ATOM H81 HA 0.09

ATOM H82 HA 0.09

GROUP

ATOM C9 CT2 -0.10

ATOM H91 HA 0.09

ATOM H92 HA 0.09

GROUP

ATOM C10 C 0.51

ATOM O1 O -0.51

GROUP

ATOM N1 NH1 -0.47

ATOM HN11 H 0.31

GROUP

ATOM C11 CT2 -0.10

ATOM H111 HA 0.09

ATOM H112 HA 0.09

GROUP

ATOM C12 CT2 0.115

ATOM H121 HA 0.09

ATOM H122 HA 0.09

ATOM N2 NH3 -0.07

ATOM HN21 HC 0.24

ATOM HN22 HC 0.24

GROUP

ATOM C13 CT2 0.115

ATOM H131 HA 0.09

ATOM H132 HA 0.09

GROUP

ATOM C14 CT2 0.21

ATOM H141 HA 0.05

ATOM H142 HA 0.05

ATOM N3 NH3 -0.30

ATOM HN31 HC 0.33

ATOM HN32 HC 0.33

ATOM HN33 HC 0.33

BOND C1 C2 C2 C3 C3 C4 C4 C5 C5 C6

BOND C6 C7 C7 C8 C8 C9 C9 C10

BOND C10 N1 N1 C11 C11 C12 C12 N2 N2 C13

BOND C13 C14 C14 N3

BOND C1 H11 C1 H12 C1 H13

BOND C2 H21 C2 H22

BOND C3 H31 C3 H32

BOND C4 H41 C4 H42

BOND C5 H51 C5 H52

BOND C6 H61 C6 H62

BOND C7 H71 C7 H72

BOND C8 H81 C8 H82

BOND C9 H91 C9 H92

BOND N1 HN11

BOND C11 H111 C11 H112

BOND C12 H121 C12 H122

BOND N2 HN21 N2 HN22

BOND C13 H131 C13 H132

BOND C14 H141 C14 H142

BOND N3 HN31 N3 HN32 N3 HN33

DOUBLE C10 O1

IMPR C10 C9 N1 O1 N1 C10 C11 HN11

DONOR HN11 N1

DONOR HN21 N2

DONOR HN22 N2

DONOR HN31 N3

DONOR HN32 N3

DONOR HN33 N3

ACCEPTOR O1 C10

PATCH FIRST NONE LAST NONE

**C.** CHARMM topology for Pep1b.

Name Type Charge (e)

RESI 　　PEP1 　　0.00

GROUP

ATOM C1 CC 0.62

ATOM O1 OC -0.76

ATOM O2 OC -0.76

ATOM C2 CT2 -0.28

ATOM H21 HA 0.09

ATOM H22 HA 0.09

GROUP

ATOM C3 CT2 -0.18

ATOM H31 HA 0.09

ATOM H32 HA 0.09

GROUP

ATOM C4 CT2 -0.10

ATOM H41 HA 0.09

ATOM H42 HA 0.09

GROUP

ATOM C5 C 0.51

ATOM O3 O -0.51

GROUP

ATOM N1 NH1 -0.47

ATOM HN11 H 0.31

GROUP

ATOM C6 CT1 -0.11

ATOM H61 HB 0.09

ATOM C7 CC 0.62

ATOM O4 OC -0.76

ATOM O5 OC -0.76

GROUP

ATOM C8 CT2 -0.18

ATOM H81 HA 0.09

ATOM H82 HA 0.09

GROUP

ATOM C9 CT2 -0.10

ATOM H91 HA 0.09

ATOM H92 HA 0.09

GROUP

ATOM N2 NH1 -0.47

ATOM HN21 H 0.31

GROUP

ATOM C10 C 0.51

ATOM O6 O -0.51

GROUP

ATOM C11 CT1 0.07

ATOM H111 HB 0.09

GROUP

ATOM C12 CT2 -0.18

ATOM H121 HA 0.09

ATOM H122 HA 0.09

GROUP

ATOM C13 CY -0.03

ATOM C14 CA 0.035

ATOM H141 HP 0.115

ATOM N3 NY -0.61

ATOM HN31 H 0.38

ATOM C15 CPT 0.13

ATOM C16 CPT -0.02

GROUP

ATOM C17 CA -0.115

ATOM H171 HP 0.115

GROUP

ATOM C18 CA -0.115

ATOM H181 HP 0.115

GROUP

ATOM C19 CA -0.115

ATOM H191 HP 0.115

GROUP

ATOM C20 CA -0.115

ATOM H201 HP 0.115

GROUP

ATOM N4 NH1 -0.47

ATOM HN41 H 0.31

GROUP

ATOM C21 C 0.51

ATOM O7 O -0.51

GROUP

ATOM C22 CT1 0.30

ATOM H221 HB 0.09

ATOM N5 NH3 -0.30

ATOM HN51 HC 0.33

ATOM HN52 HC 0.33

ATOM HN53 HC 0.33

GROUP

ATOM C23 CT2 -0.18

ATOM H231 HA 0.09

ATOM H232 HA 0.09

GROUP

ATOM C24 CT2 -0.18

ATOM H241 HA 0.09

ATOM H242 HA 0.09

GROUP

ATOM C25 CT2 0.20

ATOM H251 HA 0.09

ATOM H252 HA 0.09

ATOM N6 NC2 -0.70

ATOM HN61 HC 0.44

ATOM C26 C 0.64

ATOM N7 NC2 -0.80

ATOM HN71 HC 0.46

ATOM HN72 HC 0.46

ATOM N8 NC2 -0.80

ATOM HN81 HC 0.46

ATOM HN82 HC 0.46

BOND C1 O1 C1 C2 C2 C3 C3 C4 C4 C5

BOND C5 N1 N1 C6 C6 C7

BOND C7 O5 C6 C8 C8 C9 C9 N2 N2 C10

BOND C10 C11 C11 C12 C12 C13

BOND C14 N3 N3 C15 C13 C16 C16 C17

BOND C18 C19 C15 C20

BOND C11 N4 N4 C21 C21 C22 C22 N5

BOND C22 C23 C23 C24 C24 C25 C25 N6 N6 C26

BOND C26 N8

BOND C2 H21 C2 H22

BOND C3 H31 C3 H32

BOND C4 H41 C4 H42

BOND N1 HN11

BOND C6 H61

BOND C8 H81 C8 H82

BOND C9 H91 C9 H92

BOND N2 HN21

BOND C11 H111

BOND C12 H121 C12 H122

BOND C14 H141

BOND N3 HN31

BOND C17 H171

BOND C18 H181

BOND C19 H191

BOND C20 H201

BOND N4 HN41

BOND C22 H221

BOND N5 HN51 N5 HN52 N5 HN53

BOND C23 H231 C23 H232

BOND C24 H241 C24 H242

BOND C25 H251 C25 H252

BOND N6 HN61

BOND N7 HN71 N7 HN72

BOND N8 HN81 N8 HN82

DOUBLE C1 O2 C5 O3 C7 O4 C10 O6 C21 O7

DOUBLE C13 C14 C15 C16 C17 C18 C19 C20

DOUBLE C26 N7

IMPR C1 C2 O1 O2

IMPR C5 C4 N1 O3 N1 C5 C6 HN11

IMPR C7 C6 O4 O5

IMPR N2 C10 C9 HN21 C10 C11 N2 O6

IMPR N4 C21 C11 HN41 C21 C22 N4 O7

IMPR C26 N7 N8 N6

DONOR HN11 N1

DONOR HN21 N2

DONOR HN31 N3

DONOR HN41 N4

DONOR HN51 N5

DONOR HN52 N5

DONOR HN53 N5

DONOR HN61 N6

DONOR HN71 N7

DONOR HN72 N7

DONOR HN81 N8

DONOR HN82 N8

ACCEPTOR O1 C1

ACCEPTOR O2 C1

ACCEPTOR O3 C5

ACCEPTOR O4 C7

ACCEPTOR O5 C7

ACCEPTOR O6 C10

ACCEPTOR O7 C21

PATCH FIRST NONE LAST NONE
